# Incarcerated aboriginal women’s experiences of accessing healthcare and the limitations of the ‘equal treatment’ principle

**DOI:** 10.1186/s12939-020-1155-3

**Published:** 2020-04-03

**Authors:** S. Kendall, S. Lighton, J. Sherwood, E. Baldry, E. A. Sullivan

**Affiliations:** 1 Australian Centre for Public and Population Health Research, Faculty of Health, University of Technology Sydney, Broadway, 2007 Australia; 2 Faculty of Medicine and Health, The University of Sydney, Sydney, 2006 Australia; 3 School of Social Sciences, UNSW Sydney, Sydney, 2052 Australia; 4 Faculty of Health and Medicine, University of Newcastle, Callaghan, NSW 2308 Australia

**Keywords:** Australia, Prison, Incarceration, Women, Indigenous, Aboriginal, Health, Cultural safety, Decolonizing, Equal treatment

## Abstract

**Background:**

Colonization continues in Australia, sustained through institutional and systemic racism. Targeted discrimination and intergenerational trauma have undermined the health and wellbeing of Australia’s Aboriginal and Torres Strait Islander population, leading to significantly poorer health status, social impoverishment and inequity resulting in the over-representation of Aboriginal people in Australian prisons. Despite adoption of the ‘equal treatment’ principle, on entering prison in Australia entitlements to the national universal healthcare system are revoked and Aboriginal people lose access to health services modelled on Aboriginal concepts of culturally safe healthcare available in the community. Incarcerated Aboriginal women experience poorer health outcomes than incarcerated non-Indigenous women and Aboriginal men, yet little is known about their experiences of accessing healthcare. We report the findings of the largest qualitative study with incarcerated Aboriginal women in New South Wales (NSW) Australia in over 15 years.

**Methods:**

We employed a decolonizing research methodology, ‘community collaborative participatory action research’, involving consultation with Aboriginal communities prior to the study and establishment of a Project Advisory Group (PAG) of community expert Aboriginal women to guide the project. Forty-three semi-structured interviews were conducted in 2013 with Aboriginal women in urban and regional prisons in NSW. We applied a grounded theory approach for the data analysis with guidance from the PAG.

**Results:**

Whilst Aboriginal women reported positive and negative experiences of prison healthcare, the custodial system created numerous barriers to accessing healthcare. Aboriginal women experienced institutional racism and discrimination in the form of not being listened to, stereotyping, and inequitable healthcare compared with non-Indigenous women in prison and the community.

**Conclusions:**

‘Equal treatment’ is an inappropriate strategy for providing equitable healthcare, which is required because incarcerated Aboriginal women experience significantly poorer health. Taking a decolonizing approach, we unpack and demonstrate the systems level changes needed to make health and justice agencies culturally relevant and safe. This requires further acknowledgment of the oppressive transgenerational effects of ongoing colonial policy, a true embracing of diversity of worldviews, and critically the integration of Aboriginal concepts of health at all organizational levels to uphold Aboriginal women’s rights to culturally safe healthcare in prison and the community.

## Background

The ongoing, transgenerational effects of colonization on Aboriginal people in Australia, sustained by systemic racism and discrimination across housing, education, health and justice systems, have been effective in causing intergenerational trauma, resulting in considerable disparity in health outcomes [1–4]. The ongoing lack of respect and recognition of Aboriginal people’s rights to enjoy their own political, cultural, social and economic rights drives health disparity and inequitable distribution of resources needed for community-led approaches to support health and social and emotional wellbeing [5, 6]. Deprivation of resources that support Aboriginal people’s physical, social, spiritual and cultural wellbeing is a colonial derived practice that has been occurring with transgenerational impact since British invasion [7]. Two recent studies of the transgenerational effects of Australian government policies sanctioning forced removal of Aboriginal children from their families (known as the Stolen Generations) [1] show that Aboriginal people who are members of the Stolen Generations and their families experience increased discrimination, unstable housing, poorer health outcomes, and increased risk of imprisonment compared to Aboriginal people not of the Stolen Generations [8, 9]. These outcomes are a direct result of the intergenerational trauma caused by this ongoing political national government strategy [10]. Aboriginal people experience over-policing, harsher sentencing and a lack of basic justice that is available to the broader Australian population, resulting in their over-representation in the criminal justice system [11, 12]. The international literature has built credible evidence of such inequities experienced by Aboriginal people, including health and social inequity, discrimination and over-representation in the prison population [13, 14].

Statistical reports on Australia’s prison population further demonstrate the intersection of these inequities and the over-representation of Aboriginal people in Australian prisons. As of 30th June 2019, there were 11,866 Aboriginal and Torres Strait Islander people in prison across Australia (28% of the total prison population), compared to 31,133 incarcerated non-Indigenous people [15]. This is a vast over-representation, as Aboriginal and Torres Strait Islander people account for 3.3% of the Australian population [16]. People in prison experience a high burden of mental health and other chronic health problems [17], and more than half (57%) of people in prison in Australia have been previously incarcerated [15]. In 2018, Aboriginal people and women in prison reported poorer health outcomes than non-Indigenous people and men in prison [18]. Highlighting the negative compounding effect of incarceration and health inequity experienced by these groups, Aboriginal people and women were also more likely to report a recent history of incarceration (within 12 months) compared to previous Australian prison population surveys [18].

There was a significant rise in women’s imprisonment in Australia from 2008 to 2018, increasing by 55% compared to 29% for men, with Aboriginal women accounting for approximately one third of the women’s prison population [15]. Aboriginal women in prison in Australia have higher rates (42%) of imprisonment on remand (unsentenced) compared to non-Indigenous women (38%) and Aboriginal men (32%) [15]. Being on remand or short sentence significantly restricts access to health and other programs, limiting the opportunity to address health issues in prison and plan for release, further compounding health and social inequity [18, 19]. Surveys with Aboriginal women in prison across Australia show that they experience high levels of psychological distress, depression and anxiety connected to social and emotional wellbeing, such as unresolved trauma, removal from their families as children, and separation from their community [20–23]. Qualitative research evidence shows that this level of social and emotional dis-ease is not considered by Aboriginal women to be exceptional, rather it is the norm [24].

In New South Wales (NSW), the most populous state of Australia with the largest prison population, there were approximately 300 Aboriginal women in prison as of September 2019 compared to 660 non-Indigenous women [25]. It is important to highlight the limitations of these figures, as the number of Aboriginal women exiting prison each year (the flow population) is on average 2 times higher than the number of Aboriginal women counted in prison census (static population) figures, and even higher for younger Aboriginal women [26]. More than half of Aboriginal mothers in prison in NSW were forcibly removed from their families as children [23]. Aboriginal women in prison in NSW experience poorer health outcomes than incarcerated non-Indigenous women and Aboriginal men [20, 27]. The narratives of Aboriginal women in prison in NSW centre on the trauma of removal of their children, lack of access to secure housing on release allowing them to regain access to their children, and cycles of substance use for coping with unaddressed trauma leading to their imprisonment [24, 28, 29]. These findings correspond with international research on the experiences of Aboriginal women in prison [30, 31].

Aboriginal women in prison in NSW identify the importance and need for holistic culturally safe healthcare, housing and education for supporting Aboriginal women in prison and outside [24]. Yet, Aboriginal women (and men) in prison have restricted access to Aboriginal Community Controlled Health Organisations (ACCHOs), Aboriginal Medical Services (AMS) and government-funded (Medicare) health assessments available to them in the community [32]. Aboriginal Community Controlled Health Organisations and Aboriginal Medical Services are guided by Aboriginal concepts of health and social and emotional wellbeing, acknowledging the ongoing effects of colonization on Aboriginal people and communities [5]. They provide culturally safe continuity of care for an increasing number of Aboriginal people [33]. The Medicare health assessment item specifically established for Aboriginal people targets the early onset of chronic illnesses that disproportionately affect Aboriginal people due to the transgenerational impacts of colonization on health outcomes [34].

These restrictions on prison healthcare relate to the structure and philosophy of the Australian health system. On entering prison in Australia, entitlements to the publicly funded national universal healthcare system, Medicare, are revoked under the Health Insurance Act and access to pharmaceutical benefits is highly restricted [35]. Only 9% of Aboriginal people in prison in Australia report receiving treatment or consultation from an ACCHO or AMS whilst in prison and incarcerated Aboriginal women are more likely than Aboriginal men to report not always receiving culturally safe health care [18]. This is despite the recommendations of the *Royal Commission into Aboriginal Deaths in Custody* for Aboriginal people in prison to have access to culturally safe health care and Aboriginal-specific health services [36]. There is a body evidence that the lack of take up of the *Royal Commission into Aboriginal Deaths in Custody* recommendations has resulted in further deaths and poorer social and emotional wellbeing outcomes for the National Indigenous population [11, 12].

Peak medical bodies in Australia have stated that this system undermines the ‘Equal Treatment’ principle [37, 38]. The ‘Equal Treatment’ principle enshrined in the *United Nations Standard Minimum Rules for the Treatment of Prisoners (the Mandela Rules)* states the duty of health care practitioners to provide people in prison with the same standard of treatment as the non-prison population without discrimination of legal status [39]. Although Australia supports these rules in principle, exclusion from national healthcare funding constrains prison healthcare, culturally safe care, and continuity of care for incarcerated people with their treating clinicians including lack of access to the range of medications used in the community [37, 38]. Others have gone further to argue that this system not only deprives people of their liberty but further punishes them [40]. Responsibility for prison healthcare varies widely between the states in Australia. In some states, private providers and local public health services provide prison health services, in others healthcare is the responsibility of The Department of Corrections, and in NSW a discrete entity, the ‘Justice Health and Forensic Mental Health Network’ is funded via the NSW Ministry of Health to primarily deliver these services. There are no national standards for measuring the type and quality of services and outcomes of prison health care in Australia [41], resulting in substantial gaps in evidence to advocate for service delivery change. In addition, there has been a steady critique of the lack of attention to the subjective experiences of Aboriginal women in prison [42–45]. There is a particular dearth of evidence of incarcerated Aboriginal women’s unique experiences of accessing prison health services in Australia [24, 29, 46].

There is an Indigenous Rights and health equity imperative to address these issues. The *United Nations Declaration on the Rights of Indigenous People* states Indigenous people’s rights to their traditional health practices; enjoyment of the highest attainable physical and mental health; to develop and determine health programmes affecting them; and that particular attention shall be paid to the rights and special needs of Indigenous women and children [47]. This study aims to address this gap drawing on findings from a qualitative study of Aboriginal women in prison in NSW. We took a decolonizing theoretical approach inclusive of Indigenous research methods. We argue the limitations of ‘equal treatment’ as a guiding principle for health care for Aboriginal women in prison, as Aboriginal women do not have equitable access to healthcare in the community or in prison and require Aboriginal community controlled health services modelled on Aboriginal concepts of culturally safe healthcare. Aboriginal women utilise a variety of strategies to overcome the barriers they experience in accessing equitable and culturally safe healthcare, however, integration of trauma-informed, culturally safe models of healthcare inside and outside of prison are required to improve healthcare accessibility for incarcerated Aboriginal women. This study forms part of a larger National Health and Medical Research Council Australia project investigating the health of Aboriginal mothers in prison in two Australian jurisdictions [23].

## Theoretical underpinnings

The decolonizing approach utilised in this study draws on critical social theory and Indigenous research methods to privilege Aboriginal people’s ways of knowing, being and doing [48]. Critical social theories draw into question the positivistic claim that there is a single objective truth and go further to argue that dominant Western paradigms reproduce inequity through the production of ‘evidence’ that inherently denies or marginalises alternative points of view. Critical theoretical approaches are concerned with addressing social justice issues. From an applied ethics perspective, critical social theory points to the value of intersubjective decision-making including the perspectives of all people affected by the decisions in a discursive process [49]. For Indigenous people, Western paradigms are an integral part of colonization, subjugating and invalidating Indigenous peoples’ knowledge to rationalize and maintain the colonial agenda. Western research contributes to this agenda through employment of methods that exclude consultation with Aboriginal people and communities and, consequently the proliferation of deficit understandings of Aboriginal people [48, 50]. We sought to decolonize these ways of doing research, collaborating with Aboriginal women and their communities, acknowledging them as the experts on their needs [51].

Methodologies used in research for surveillance of prison population health lead to problematic understandings of incarcerated Aboriginal women due to the lack of attention to context, including lack of acknowledgement of the ongoing effects of colonization on Aboriginal women and their communities and lack of attention to issues of health equity and women’s subjective experiences [19]. Our decolonizing approach acknowledges the ongoing effects of colonization on Aboriginal health, and the variation of the effects of colonization in different locations across Australia, guided by Aboriginal concepts of health and social and emotional wellbeing [51, 52]. We used a reflexive, iterative and intersubjective approach to the construction of knowledge in collaboration with Aboriginal women and their communities.

Prior to the data collection, the lead Aboriginal researcher in NSW and other members of the research team spent a number of years consulting and working with Aboriginal women’s community organisations across NSW, forming a Project Advisory Group (PAG) that guided the research throughout all stages of the study. When the data collection began, Aboriginal women in prison were supported in telling their stories of resilience. We interpreted the data through a process of dialogue, shared understanding, and collaborative consensus with the women who participated and the PAG. In this article, we have focused on what women said about their experiences of the prison health system and healthcare accessibility in prison and outside. We took an inductive, grounded theory approach to the data analysis to develop themes centred on what the women said. The aim of this research is to elucidate incarcerated Aboriginal women’s experiences of prison healthcare, investigate equity of access to culturally safe healthcare in prison, and identify pathways for improving the accessibility of culturally safe healthcare.

## Methods

The EQUATOR Standards for Reporting Qualitative Research were used in the preparation of the manuscript [53].

### Methodology

This project was guided by a community collaborative participatory action research methodology [52] and a grounded theory approach [54]. Community collaborative participatory action research is an iterative, multi-methods approach positioning Aboriginal people as the experts [48, 51, 52]. In this project, collaboration with Aboriginal women and their communities guided all stages of the project from planning through to dissemination, including 2 years of consultation preceding the data collection. This approach ensured that the aims and methods of the study aligned with community priorities. The two-year consultation stage led to the formation of the PAG to guide the remaining stages of the research with members from Aboriginal community organisations, Aboriginal community controlled health services, government departments (corrective services and justice health) and non-government organisations. We developed the data collection tools in collaboration with the PAG and held regular meetings during the analysis to collaborate on interpretation of the data. Aboriginal concepts of health and social and emotional wellbeing framed our research, recognising the importance to Aboriginal people of connection to land, culture, spirituality, ancestry, family and community. This view of health requires looking beyond the physical needs of the individual and understanding health in a context of inequity, trauma, racism and discrimination and the influences of what assists people in accessing health services [3]. The relationship with the PAG is ongoing.

### Recruitment

We employed a purposive, convenience sampling method, including NSW prisons housing 10 or more women. Prisons with less than 10 women were excluded from the sample. Six prisons were included in the sample. A combination of minimum, medium, and maximum security prisons located in urban and regional locations in NSW were included. This was the most appropriate sampling method for involving as many women in the research as possible given the vast geographical distances between prisons in NSW, and the logistical challenges of prison research including unexpected transfer of women between prisons, release of women to the community and security ‘lock-downs’ blocking access to the women.

We built respectful relationships with participants and their communities prior to the data collection, including prison visits to share information about the project, and have maintained these beyond the funded project. Data collection in NSW was conducted in two stages, the first stage involving a participant-administered health and social and emotional wellbeing quantitative survey, with recruitment open to all Aboriginal women in prison. The findings of the survey are reported elsewhere [23]. This first stage of data collection gave the researchers the opportunity to get to know the women and invite them to participate in the qualitative stage of the research. As this study was part of a wider study investigating the health of Aboriginal mothers in prison in Australia, the qualitative component of the research focused on the experiences of Aboriginal mothers in prison. The vast majority of women in prison are mothers [18]. Inclusion criteria for the interviews were that the women self-identified as Aboriginal and as a mother. Participation was voluntary and women were advised that they could withdraw from the research at any time. All participants provided informed consent.

### Data collection

Interviews were conducted in 2013. The lead Aboriginal female researcher in NSW and a non-Indigenous female researcher co-facilitated the interviews. Interviews were conducted in English, the primary language spoken by the women. We used a semi-structured interview guide, developed in collaboration with the PAG, and applied a yarning approach, providing space for women to tell their story through a supportive, co- facilitative method that privileged their knowledge as expert [55–57]. The interview guide contained topic areas relating to the women’s experiences of prison and health care experiences, the impact of incarceration on family and community, and the women’s goals, strengths and support systems. Sociodemographic data were not collected from interview participants, as they had already been asked to provide this data at the time of participating in the health and social and emotional wellbeing surveys. The interviews were audio-recorded and professionally transcribed. The average interview length was 30 min.

### Data analysis

We conducted a thematic data analysis [58], taking an inductive semi-grounded-theory approach to generate themes across the interviews [54]. This was a four-stage process. Stage one involved Aboriginal and non-Indigenous researchers from the project team coding and interpreting a sample of the data and developing a coding frame. As a process of internal validation, we continued coding until reaching consensus that we were identifying codes and interpreting the data consistently. Stage two consisted of two researchers from stage one coding the remaining transcripts. NVivo 10 QSR International software was utilised as a data management tool. As new codes emerged that did not appear in the coding frame, these were discussed amongst the research team. Stage three involved workshopping the codes into themes and presenting them to the PAG for interpretation and as a process of external validation. Themes were conceptualised, contextualized, and developed further by the PAG. Stage four involved reviewing and revising themes incorporating the PAG advice and finalising a conclusive set of themes and sub-themes that mapped our data. Following Braun and Clarke (2006) [58], we considered not only the story being told within each theme but how the themes interrelated and fit into the broader story told by the data.

## Results

Forty-three Aboriginal women were interviewed. This is the largest qualitative study of Aboriginal women in prison in NSW in 15 years [29]. Table 1 shows the location of participants and prison security level.

**Table 1:** Participant location and prison security level

Interview no.	Prison location	Prison security level
1–6	Urban	Minimum
7–13	Urban	Minimum/Medium
14–22	Regional	Minimum/Medium/Maximum
23–27	Regional	Minimum/Medium
28–32	Urban	Minimum
33–43	Urban	Minimum/Medium

The analysis identified four primary themes in relation to women’s experiences of accessing prison health care: 1) mixed experiences; 2) loss of autonomy; 3) institutional racism; and 4) strategies for accessing healthcare.

### Theme 1: mixed experiences

Aboriginal women described mixed experiences of prison healthcare. Some women in the study reported prison healthcare systems improved the management of some physical health conditions because the health service was on site, providing regular monitoring of health conditions, scheduled appointments and reminders. Some women who experienced improved management of their health reported positive engagement with prison health workers. For example, the woman quoted below spoke about daily follow up appointments after dental surgery for a painful abscess and attentive and thorough post-surgery care from nurses. She was no longer in pain, attributing this outcome to the healthcare she had received.

*I see a nurse every day. I went and got three teeth out last week, and I am so glad because I have an abscess on my tooth and I come in with it. I had an appointment Thursday anyway so I got them out last week anyway, which was good. No more pain. They're keeping a close eye on me because I've got community acquired MRSA [MRSA infection] and they've been turning into ulcers lately, and they're thinking whether that could be diabetes early stages or something like that there, because it takes quite a while to heal. But they've been checking my blood sugar, my sugar and all of that. (Interview 20)*

Other women in our study associated improvements in their health due to taking the correct medication in prison. These improvements in health were identified as situational, associated with effective medication management in prison, rather than particular attributes of health service providers. One woman said her epilepsy was well-managed in prison due to her taking the correct medication and not substituting with illicit drugs for symptom relief:

*I had disability pension, I've got epilepsy, so - Facilitator: Is that managed? Do you feel like it's managed well here? Interviewee: In gaol it is. The last two years I haven't had a seizure in gaol, which is good. Outside when I don't take my medication, I'm using heroin, it's - I don't usually have seizures outside because the heroin is a downer, it does calm my brain but it's not the right medication I should be taking. (Interview 35)*

Another woman spoke about improvements in her mental health due to taking regular medication in prison, although there were limitations to this. This woman told us that she was taking anti-depressant medication regularly in prison, the same that she had been prescribed in the community. In the community, she said there was no point in taking the medication because her social circumstances were the reason she was unwell *“I was still living in it”*. She reported that taking anti-depressant medication regularly in prison had made a positive difference but that she is still unwell, *“I’m still living in it”*, suggesting that the factors contributing to her social and emotional wellbeing remain unsolved. As she said:

*Facilitator: And are you well physically? Interviewee: No. No, I'm depressed. I'm on medication for it. Facilitator: Okay. Were you on that before you came inside? Interviewee: Yeah, but I shouldn’t have been taking it but because I'm still living in it, it's not worth taking anti-depressants, yeah, because I was still living in it, yeah. Facilitator: And now you're on them you can - you're taking them in here? Interviewee: Yeah, got to take them every day. Facilitator: Have they made a difference for you? Interviewee: Yeah, they have. (Interview 30)*

This woman’s story highlights the limitations of pharmacological treatments when women do not receive support for their social and emotional wellbeing or are provided comprehensive information about the medication they are taking including contraindications. We return to this issue in the discussion.

### Theme 2: loss of autonomy

There was a strong theme relating to negative experiences of loss of autonomy in the prison environment. Being in prison substantially took away women’s self-determination as health service users. Aboriginal women reported a lack of being able to participate in their care in prison and exclusion from decision-making processes about their treatment. Prison restricts the typical everyday healthcare choices taken for granted in the community, such as being able to self-diagnose and obtain over-the-counter medication at a pharmacy. Previously simple decisions about non-prescription medication, such as the use of headache medications, required approval. Women expressed feeling helpless and frustrated that they were forced to seek help for very minor conditions. Women also lost control over being able to continue some medications that had worked for them in the community and the dosage of the new medications prescribed. As said by these women:

*You know, you can't get a Panadol without seeing a doctor. There are women suffering from drug taking behaviour, I understand that but it's not everyone. (Interview 3)*

*I'm on Seroquel. I've been taking - all my information is legit. They're not giving my medication in here. I just recently come down from 1200 to 50 milligrams. (Interview 34)*

*Yeah, I suffered bad anxiety and I'm on anxiety medication outside but obviously they don't give it here. Yeah, very bad anxiety. (Interview 12)*

This lack of medication could have dire consequences. One woman shared a story with us about another woman taking her own life because she was not given the medication she needed in prison. Women also expressed loss of autonomy in relation to accessing healthcare when they needed it. Women shared experiences of long wait times for health checks, diagnostic testing, and medication review due to the workload of prison health staff. The quotes below illustrate the women’s loss of control in the face of long waiting times:

*I come in, what, late January and it come to February and I said to one of the nurses, I said, "Can you organise for me to have a Pap smear test?", and I'm still waiting on it. (Interview 27)*

*They [Anti-anxiety medication] get given to me every day … Oh, they reckon they were going to do that [review dosages] ages ago, but no, they're just too busy.* (*Interview* 15)

*I was supposed to be on antidepressants, I'm still waiting. I've been off them for about a week now. (Interview 20)*

For some women, waiting times had a profound and devastating impact on their health. Some interviews prompted the researchers, with the women’s consent, to notify the senior Aboriginal (non-custodial) staff member supporting our project at the prison sites that women needed to be seen urgently by prison health services due to sudden and substantial reductions in medication or because they were not receiving any medication. In the quote below, one woman reports two health crises triggered by lack of timely, adequate healthcare. This woman experienced a seizure after waiting 9 h for a health check in prison reception, resulting in an emergency response and transfer to the hospital. On return to the prison, she spent one night in the prison health clinic. Shortly after discharge from the clinic, she overdosed on pills in her cell, and was taken back to hospital.

*I come here, we got off the truck at 6:00, 6:30 in the morning. We got kept in one of them cells over there with just the bench silver seats. From that time in the morning until 3:30 in the afternoon we were waiting to see the nurses and then welfare to get screened before you come in. Yeah, I ended up having a seizure and that's when all of them come running. I went to the hospital … I stayed in there. They tried getting blood and that out of me, checked me over and that. Couldn't get blood. Even tried to go in my groin here and still couldn't get blood, so I was sent back here. Then when I was in here I spent one night over in the clinic and then when I come straight over here I come in with tobacco and that and I ended up swapping whatnot and getting my hands on pills from in here, so I OD'd [overdosed] the next night. So they took me back to the hospital for that. But after all that happened I've only been taking my own medication.* (*Interview* 22)

As described in the quote above, this woman experienced two life-threatening emergencies due to a failure of both the custodial and health system to ensure her safety. In the first instance this woman was placed in a life-threatening situation due to the time she had to wait for a health assessment. In the second instance, inadequate supports were in place as she transferred to a cell post-discharge from prison and community health services. Exiting care can be a time of elevated vulnerability without adequate supports [59, 60], yet in both instances for this woman, access to care required the onset of acute symptoms that could have resulted in a death in custody.

In the example below, one woman describes waiting five-months to see a psychologist (psychology services are provided by the department of corrective services in NSW rather than the justice health service), following the removal of her newborn son from her care. This woman describes reaching ‘breaking point’ during this time, as prison guards repeatedly ignored her requests for help and she began to worry that she would become suicidal. This woman had no option but to continue to report her symptoms and wait.

*After I had my son they said I had to see someone a week after I had him. I had one night and one day with him and, yeah, took me five months to actually see a counsellor. I've only seen a psychiatrist and that was only 20 minutes. There's not enough support at all, like there really isn't. It just takes forever, like I've had to ring and say that I'm not well, like I'm really depressed and I'm going to find myself in trouble because the guards don't want to listen, nobody wants to listen, I've got to break before I see someone. (Interview 4)*

This woman’s story shows not only a loss of autonomy because of her dependence on prison guards for access to healthcare and loss of rights to complaint and recourse, but also a severe and punitive lack of care for this woman who had experienced the significant trauma of her child being removed and was proactively asking for support. Custodial staff were highly negligent in their responsibility to treat this woman with respect for her dignity and wellbeing in accordance with the basic principles for the treatment of prisoners [61]. As shown by the results in the next section, for Aboriginal women, experiences of not being heard or responded to were connected with numerous experiences of institutional and individual racism.

### Theme 3: institutional racism

Aboriginal women reflected they did not receive the same treatment as non-Indigenous women in prison or in the community. Women spoke about stereotyping, being judged prejudicially, and receiving differential poorer treatment compared to non-Indigenous women. Entry into prison marked and enhanced the impact of the continuation of racism, discrimination and stereotyping in the community layered with the additional stigma of an ‘offender’ identity. As this woman said:

*We get judged a lot and I know that Aboriginal girls get judged a lot. It's stereotyping, you know, and it's like: Oh you're back in, yeah. It's not right... I've seen it. I've seen the girls in here get treated differently because they're Aboriginal and I think we all should be treated equally. I'm a big believer of that. (Interview 37)*

Notwithstanding the fact that prison provides a number of treatment options for substance use ranging from symptom relief to opioid substitution, Aboriginal women also experienced stereotyping as active drug seekers. Women described that healthcare decisions were based on this stereotype, rather than on their actual symptoms or requests, and they consequently were not able to access healthcare for the same health problems as non-Indigenous women. The following quote is illustrative, with this woman describing being accused of seeking pain medication when she had not asked for it, being called a ‘drug chaser’ by prison health staff and not being provided with any treatment for her health problem. She raises the issue of systemic discrimination, reporting that a non-Indigenous woman with the same health problem had been taken to the hospital.

*Interviewee: And there's other girls that go up there they've had bad pains in the belly - I went up there and she said I'm not giving you buscopan. I said I haven't even asked for it and why not? Why am I different to other people in here? Facilitator: What do you think that's about? Interviewee: They're just discriminating against me I think. Facilitator: Do you see everyone else getting good health service? Interviewee: Yes. Facilitator: Do other mob get it okay? Interviewee: Yes, seems like it. There's another girl in here, I don't know if it has anything to do with culture or not but she is white, and she's been taken to the hospital a lot and anything that's wrong with her they take her straight up. I've had the same things wrong with me and nothing's happened. I'm a drug chaser under their eyes. They've told me that. (Interview 15)*

There is evidence that this framing of Aboriginal women’s symptoms and signs can result in denial or delay in access to culturally safe health care as seen with the recent high profile case of Ms. Dhu, an Aboriginal woman who was in police custody for un-paid fines in Western Australia. Ms. Dhu was prejudicially labelled by health and custodial staff as seeking drugs, although she was in severe pain and eventually died of cardiac failure. The coroner in this case reported the death of Ms. Dhu was due to deficient hospital treatment [62]. Women in our study also reported experiences with the medical profession outside of prison where they were not adequately attended to due to stereotyping. In the quote below, one woman describes going to hospital after an incidence of intimate partner violence where her injury was treated as an accident and not reported.

*Well on the day of the incident I was knocked out unconscious and the doctor that - I woke up in hospital, he was stitching me up, so he didn't know, he wasn't aware that it was - it was an attack. He thought it was just an accident and he let me out of hospital. And I'm remembering more - I was remembering more because I moved out of A [place name removed] and went to B [place name removed] and used hot water, hot baths to give me relief and the doctor down at A [place name removed] wasn't aware that I was actually attacked and beaten and bashed by this [partners name removed]. And he just thought it was an accident, stitched me up and let me out. (Interview 33)*

This experience of the woman quoted above is consistent with the concerns raised in Australia’s recent address to the United Nations Human Rights Council in response to the Special Rapporteur on Violence Against Women, which highlight that the majority of violence against Aboriginal women goes unreported and women are “too often met with systemic racism when they reach out for help” [63]. Our study shows that racism within the custodial and health systems can result in exacerbation of health problems, inadequate healthcare, and blocked access to healthcare for Aboriginal women.

### Theme 4: strategies for accessing healthcare

The women’s narratives evidenced a strong belief in the importance of health matched with pro-action regarding their health. However, because the prison healthcare system contains unique procedures, rules and power relationships, some of which become known to the women over time and some of which continue to be opaque, women had to employ a variety of strategies to access prison health services. Women who were new to prison reported a lack of orientation to the prison healthcare system. These women reported difficulty in obtaining information to access health services and frustration with the lack of efficiency within the system. In the absence of induction to the system and established peer-networks to facilitate learning about the system, custodial officers were gatekeepers to the prison health service. However, interactions with custodial staff could be a barrier to accessing healthcare, as illustrated by the following quote:

*I did a green form and he laughed at me. He goes, you don’t do a green form. See, because I'm new, I … no-one's explained anything. That's another thing that I don’t like, is they don’t explain anything. (Interview 19)*

Women who had been in prison for some time or who had been imprisoned previously typically had a better knowledge of the operational dimension of the prison healthcare system. These women were able to work with the system to engage with health services. They knew which forms to fill in and where to submit them. They had knowledge of which staff member would be most appropriate to speak to regarding their needs and held an appreciation of the likely timeframes for treatment. Some women wanted to build positive relationships with prison officers to enhance their engagement with services and programs; others were simply pragmatic about their engagement with the system. As these women said:

*Yeah, I try to be encouraging. Like if I do end up doing – if I do get a custodial sentence and I do, like, you know two years or something, I want to actually really get in a good relationship with the officers out here and get a lot of programs with, you know, educational programs for, you know, HIV and sharing needles and stuff. (Interview 39)*

*When I first come in last Monday, before you come in here, you see a nurse and they ask you if you've got anything wrong with you and I said yeah I've got an ulcer and a boil coming up, and they look at it and check it, maybe swab it. They go on from there, and because I've had it before and because it needs clean dressings, it's required to keep it under check because it can easily spread. So I just go in, and if they don't call me up you just go sick in the cell. You can only do that in the morning or you put in a green form with the nurse that comes in to hand out the pills and say that you need to see a nurse ASAP [as soon as possible] for such and such reason. So you get called up then, if it's urgent. (Interview 20)*

Some women described needing to challenge the system to access healthcare. Women adopted this position as a result of failed attempts to engage with the system on a more positive basis. Challenging the system involved voicing their dissatisfaction and unmet needs. Some women challenged the diagnosis and treatment they had received in prison. As shown by the quotes below, others challenged system inefficiencies, inconsistent information, lack of follow up, and the process for accessing the Methadone program.

*I'm coming here every day going can I have a request form, you told me to go here after 4.30, they go no, we told you to go here. I come back at 3.30 the next day, right, they go no, you've got to be here after 4.30. I said is it 3.30 or 4.30? I said, because different times, what time do I come in here and what day? And I thought why is it so hard, why isn't there a direct time to get a bloody request form. You have to be there at a certain time and speak to a certain officer on a certain day. Like I say, it's all about management - managing appointments, you know, and stuff like that but they have no sense of management … the staff doesn't know what they're doing, how are we supposed to know what we're doing, you know what I mean? (Interview 4)*

*I've seen the Methadone doctor three times and all he did was put me on Neulactil and he said - because I'm on the Avanza and whatnot that I'm on enough medication. I'm still waiting for the methadone. I've put two green forms in and I haven't heard nothing back yet, so I don't know. They're not good with that I don't think since they've changed all the rules. You used to be able to get on it when you come in but now all your paperwork's got to be sent to Sydney and you go on a waiting list. (Interview 22)*

The lengths that women had to go to in order to get answers to simple questions about forms and timeframes for accessing healthcare, only to be met with incorrect answers and inconsistent information from staff, is highly problematic considering the health risks in the environment and that many women come into prison seeking healthcare. Long waiting times, the difficulties Aboriginal women in prison experience in getting custodial staff to treat their requests for help as legitimate, and experiences of discrimination from health service providers resulting in their health concerns going unaddressed, illustrate an inaccessible, culturally unsafe health system for Aboriginal women in prison.

## Discussion

Our findings show that Aboriginal women in prison experience multiple barriers to accessing culturally safe healthcare. The prison health system is discrete from the universal healthcare system accessed by women in the community and difficult to navigate. Navigating the system is challenging because processes are dictated by custodial regimes and are not transparent or consistently implemented. Women experience a loss of autonomy and many of their rights as health service users when they enter prison principally because the remit of the custodial system to maintain security takes precedence over the accessibility of the prison health service [64]. Custodial staff hold a great deal of power as they are in positions of authority that allow them to judge women’s requests for healthcare as legitimate or not. Further, the prison health system is not structured in a way that it is possible for the prison health service to provide equal treatment to what is available in the community due to the exclusion from national healthcare funding including subsidies for pharmaceuticals. Whilst some women in our study reported improvements in the management of health conditions and benefit from taking regular medication in prison, the vast majority of women shared experiences of not having their health needs met.

In the process of accessing healthcare, women in this study reported discontinuation of effective medications on entry to prison, abrupt reductions in medication and excessive wait times for assessment and medication review. Prison health service providers did not adequately inform some women about new medications and diagnoses, creating a critical dilemma for these women when they leave prison and are trying to sustain their wellbeing outside. Women lost the option to manage their health problems, and at the same time could not access healthcare when they needed it. Some women had to be transferred to local hospitals due to the onset of acute symptoms while they waited for prison healthcare and did not receive adequate follow up or support on their return, triggering a rapid deterioration in their mental state and instances of self-harm. For some women, not being able to access healthcare when needed was attributed to the workloads of prison health staff and general waiting times. Other women described their unmet health needs as resulting from custodial and health staff disregarding their requests for healthcare. These women reported racism and discriminatory treatment and quality of care from custodial and prison health staff in the form of judgementalism and stereotyping, resulting in them not being listened to or taken seriously, having their symptoms ignored or misread, and not being offered the same treatment as non-Indigenous women for the same health issues. Women experienced punitive treatment when they asked for help such as being ignored whilst their health deteriorated, being ridiculed for not knowing how to navigate the system and being told inconsistent information about how the system works. This treatment is in contravention of the human rights of Indigenous people [47] and the recommendations of the *Royal Commission into Aboriginal Deaths in Custody* [36] that Aboriginal people have equitable access to culturally safe healthcare. It also shows that responsibilities as outlined in the *Basic Principles for the Treatment of Prisoners*, particularly those pertaining to treating prisoners with respect for their dignity and worth, promoting their wellbeing, and respecting cultural values [61], were not upheld.

For women in this study, failure to uphold these principles led to a worsening of symptoms, compounded distress, and life-threatening health crises. Some women described becoming suicidal after requests for help were repeatedly ignored by custodial staff. Due to the loss of their patient rights to complaint and resolution, these women had no choice but to persevere with staff members who continually discredited the legitimacy of their requests. Other scholars have argued that the systems effecting Aboriginal people in prison are ineffective in delivering equitable mental health care partly due to the lack of understanding and poor handling by staff of issues such as grief, disconnection, discrimination and intergenerational trauma [65, 66]. Our study reinforces this claim showing how interactions with prison staff sustain and compound intergenerational trauma and health inequity by failing to recognise when Aboriginal women are distressed and denying Aboriginal women healthcare when they seek help. Aboriginal women in our study reported feeling as though they ‘have to break’ before they will get any support.

These findings reveal significant limitations of ‘equal treatment’ as a guiding principle for addressing health disparity [67]. The health disparities experienced by Aboriginal women in prison are rooted in inequity and racism across numerous systems. The healthcare Aboriginal women received in prison was culturally unsafe, and in this way not equal to that received by non-Indigenous women in prison. Likewise, Aboriginal women did not receive ‘equal’, culturally safe healthcare compared with non-Indigenous women in the community. Previous research shows that Indigenous people experience disparate healthcare treatment and quality [68–71]. Our study provides further evidence that inequitable access to culturally safe healthcare and institutional racism reinforces and results in further health disparities and negative physical and mental health outcomes [72–74].

Taking a decolonizing approach, we argue that colonization continues to reduce any form of equitable and culturally safe treatment that Aboriginal people can expect from government services including perpetuating health disparities in and out of prison. It is a concern that Aboriginal concepts of health remain largely tokenistic within the prison and wider health systems, especially in light of the recommendations from the *Royal Commission into Aboriginal Deaths in Custody* for culturally safe health services. Systemic marginalisation of Aboriginal knowledge and norms by systems of Western governance are drivers of culturally unsafe healthcare. Non-Indigenous healthcare professionals typically perceive patient factors as the underlying cause of healthcare disparities, rather than their own cultural biases, social factors or systemic discrimination [75]. This creates a situation where responsibility for the disparity of health outcomes is shifted onto patients, and in a self- perpetuating cycle, patients are stigmatised and not listened to, contributing further to health disparity and inequity [75]. Similar patterns of power and control have been identified in a study of residential programmes for mothers and children in Australian prisons, revealing that Aboriginal mothers experience a punitive, paradoxical situation where they are at the same time held responsible, disempowered and highly scrutinized for their parenting skills based on Western concepts of mothering [76].

The ‘equal treatment’ principle asserts that people in prison should be provided the same quality of healthcare treatment as people in the community. What is not taken into account is the inequity of access to culturally safe healthcare experienced by Aboriginal women because Aboriginal concepts of health are not equally embedded in mainstream health services alongside Western concepts of health [66]. Aboriginal women hold holistic and collective views of health connected to family and community social and emotional wellbeing and prioritise the collective rights of the community over individual rights [77]. Aboriginal women in our study highlighted the limited benefits of Western knowledge and medical approaches, particularly pharmacological treatment of mental health problems, when they receive no support for their social and emotional wellbeing and inadequate information about their diagnosis or medications by prison health services. The Aboriginal concept of social and emotional wellbeing is different from the Western concept of mental health, encompassing the effects of ongoing colonization and unresolved trauma. It is defined as follows [78]:

*“the social and emotional wellbeing concept is broader than this and recognises the importance of connection to land, culture, spirituality, ancestry, family and community, and how these affect the individual. Social and emotional wellbeing problems cover a broad range of problems that can result from unresolved grief and loss, trauma and abuse, domestic violence, removal from family, substance misuse, family breakdown, cultural dislocation, racism and discrimination and social disadvantage” (Social Health Reference Group, 2004, page 9).*

While healthcare systems continue to operate on Western concepts of health centred only on treating illness within the individual, responsibilities in the provision of ‘equal treatment’ will be confined to, at best, healthcare providers offering Aboriginal women in prison what they would have offered them in the community. Recognising that Aboriginal women often do not receive good treatment in the community unless from an Aboriginal community controlled health service [79], particularly Aboriginal women who have experienced incarceration [46], denial of access to these services deprives Aboriginal women in prison of equitable access to culturally safe healthcare. Communities also need to be resourced to extend the capacity of Aboriginal community controlled health services to provide the equivalent Aboriginal health service to Aboriginal women in prison as in the community, and other services to support the social and emotional wellbeing of Aboriginal women exiting prison [6].

In terms of the aim of this study to identify pathways for improving the accessibility of culturally safe healthcare, we propose improved access to Aboriginal community controlled health services in prison alongside improvements in the cultural safety of Australian healthcare and other systems. Cultural safety is a strategy driven from an Indigenous agenda to unpack the impact of colonization and the health system’s lack of respect for First Nation Peoples’ ways of viewing health and wellbeing. Respect and equivalence of Aboriginal and Torres Strait Islander knowledges is vital to Aboriginal and Torres Strait Islander health and addressing the health disparity and significant inequity in healthcare experiences of Aboriginal and Torres Strait Islander people [75, 80]. The focus of cultural safety is to decolonize the health service inertia, which has failed to address the systemic, institutional and individual acts of racism known to cause health inequalities in healthcare provision [80]. Ideally, this should improve access and the quality and effectiveness of care.

### Limitations

The findings of this study are unique to the Aboriginal women we interviewed and may not be generalizable to Aboriginal women in other jurisdictions. However, the convenience sampling technique we used was the most appropriate method for including as many Aboriginal women in the study as possible and resulted in the largest qualitative study of Aboriginal women in prison in 15 years [29]. Further, the aim of this study was to respect the experiences of Aboriginal women in NSW, acknowledging that there are differences in the histories and transgenerational effects of colonization on communities across Australia. Our study was an iterative process conducted in collaboration with the project advisory group of expert Aboriginal women from NSW following Indigenous research protocols.

## Conclusion

Statements about the priorities of prison healthcare in Australia recognise that Aboriginal women in prison experience health and social inequity and explicitly articulate commitment to the equal treatment principle and the need for practitioners to provide high quality, culturally safe health care that improves health outcomes [38, 81]. Our study shows there are significant limitations to the ‘equal treatment’ principle for addressing the inequity of access to culturally safe health services for Aboriginal women in prison and outside. A decolonizing, cultural competence approach including enhanced access to Aboriginal community controlled health services in prison is required to address this inequity. The NSW prison health service recently stated the need to address the disparity of health outcomes experienced by Aboriginal women and appointed a family support worker from ‘Waminda South Coast Women’s Health and Welfare Aboriginal Corporation’, the only Aboriginal Community Controlled Health Service in NSW specifically for women, to provide in-reach support to Aboriginal women in prison returning to the South Coast catchment area. This unique program was not available to Aboriginal women at the time of this study and future research should seek to speak to Aboriginal women accessing this culturally safe service about their experiences to inform further policy and practice change.

## Data Availability

The dataset analysed during this study is not publicly available due to ethics requirements.

## Abbreviations

ACCHOs: Aboriginal Community Controlled Health Organisations
AMS: Aboriginal Medical Services
NSW: New South Wales
PAG: Project Advisory Group
